# Development and validation of a nomogram prediction model for hypertension-diabetes comorbidity based on chronic disease management in the community

**DOI:** 10.1186/s12944-023-01904-1

**Published:** 2023-08-24

**Authors:** Yan Wu, Wei Tan, Yifeng Liu, Yongli Li, Jiali Zou, Jinsong Zhang, Wenjuan Huang

**Affiliations:** 1 Geriatric Hospital Affiliated to Wuhan University of Science and Technology, Wuhan, 430081 China; 2 Psychological Depression Ward, Wuhan Mental Health Center, Wuhan, 430012 China

**Keywords:** Hypertension-diabetes comorbidity, Chronic disease management, Nomogram, Prediction model, Community health service center

## Abstract

**Purpose:**

​Develop and validate a nomogram prediction model for hypertension-diabetes comorbidities based on chronic disease management in the community.

**Patients and methods:**

The nomogram prediction model was developed in a cohort of 7200 hypertensive patients at a community health service center in Hongshan District, Wuhan City. The data were collected from January 2022 to December 2022 and randomly divided into modeling and validation groups at a 7:3 ratio. The Lasso regression model was used for data dimensionality reduction, feature selection, and clinical test feature construction. Multivariate logistic regression analysis was used to build the prediction model.

**Results:**

The application of the nomogram in the verification group showed good discrimination, with an AUC of 0.9205 (95% CI: 0.8471–0.9527) and a good calibration effect. Decision curve analysis demonstrated that the predictive model was clinically useful.

**Conclusion:**

This study presents a nomogram prediction model that incorporates age, waist-height ratio and elevated density lipoprotein cholesterol (HDL-CHOLESTEROL), which can be used to predict the risk of codeveloping diabetes in hypertensive patients.

## Introduction

​The number of chronic diseases is gradually increasing as the aging process of the population accelerates and the dietary structure of residents changes following improvements in living conditions, and chronic diseases have become the top threat to national health. ​In the Special Report of the Sixth National Health Service Statistical Survey, 48.4% of people aged 55 to 64 and 62.3% of people aged 65 and over had a chronic condition. Hypertension and diabetes are common chronic diseases with steep rates. It has been reported that the number of elderly people suffering from hypertension has reached 250 million [1], which poses a enormous challenge to the prevention and treatment of chronic diseases. It has been shown that the incidence of hypertension is positively correlated with the cultural background and cognitive level of the patients, such as agricultural household registration and low cultural education, and the risk of hypertension is significantly increased, suggesting that in primary care. It is necessary to focus on the prevention and control of hypertension in rural and remote poor areas, especially for those with limited education and poor information [2].​ Another survey shows that the awareness rate of hypertension in China is only 61%, and the control rate continues to hover at a low level (< 50%). ​

Therefore, how to implement effective diversified health education for every primary care staff member is worth considering. By actively improving the awareness of residents, we can help reduce the incidence of hypertension, thereby reducing the risk of cardiovascular diseases [3]. Among the administered population in this jurisdiction, the coincidence rate of hypertension and diabetes was approximately 6.04%, which was much lower than other survey results (11.25%) [4]; ​however, the coincidence rate of hypertension and diabetes was as high as 10.27% in women over 75 years of age and showed clear signatures of elevated cardiovascular disease incidence. Therefore, we should focus on dynamic glucose monitoring and health promotion and education for hypertensive patients to reduce the incidence of hypertension-diabetes comorbidities. ​The low incidence of HDC in this jurisdiction compared to the results of other studies may be related to the fact that there are four colleges and universities in this jurisdiction, and the majority of college students are healthy young adults. It may also be related to objective factors such as excess diabetes and differences in the distribution of the sample population.

​Dyslipidemia is an essential risk factor for hypertension and HDC, and enhanced daily lipid monitoring is particularly critical in this population. The risk of cardiovascular disease and death can be significantly reduced through the quantitative management of lipid levels [5]. Among the hypertensive population in China, the incidence of dyslipidemia is as high as 42.7%. However, only 20% of patients receive timely clinical treatment for dyslipidemia. The main reason is that health managers have insufficient understanding of the correlation between dyslipidemia and chronic disease progression and have no active awareness of monitoring blood lipids in daily chronic disease management [6]. ​Therefore, we need to not only strengthen normal health education in high-risk groups with dyslipidemia but also monitor the dynamic changes in blood lipids and implement effective lipid management in a timely manner to reduce the risk of cardiovascular disease.

A common feature of prehypertensive patients and prediabetic patients is insulin resistance, and insulin resistance is an early stage that can develop into two different diseases [7]. Studies have shown that obesity is the main cause of insulin resistance [8]. In foreign countries, most current research has focused on the correlation between obesity indicators and individual hypertension or diabetes. Domestic reports have shown a positive correlation between obesity indicators and HDC. For example, Liu et al. studied 10,431 adults in some areas of Jiangsu and Anhui, Fu et al. focused on adults in Beijing, and Xie et al. studied 24,792 residents in Chongqing who were 40 years old and concluded that obesity increased the risk of HDC. Garrow et al. proposed that the use of BMI to predict obesity has limitations on the risk of cardiovascular diseases [9]. ​Although the indicator WHtR is not widely used, it is more accurate than BMI and WC in predicting HDC risk. The findings of this study suggest that BMI and WHtR are independent factors in the risk of hypertension-diabetes comorbidity.

Based on the above, it is necessary to include BMI, WC, WHtR and other indicators in the analysis of influencing factors of the HDC of residents in jurisdictions, use a new method to describe the change trajectory of their respective association intensity with the HDC of residents in jurisdictions, and further analyze their relationship with the HDC of residents in jurisdictions to judge their respective prediction efficiency of the HDC risk of residents in jurisdictions. It would also be of strong practical interest if the population studied were targeted at the residents of the jurisdiction.

Nomogram prediction models are methods used to assess the probability of occurrence of certain diseases. Due to its superior reliability, it has been widely used in cancer and other research fields [10–12]. It can not only predict the risk of disease occurrence and gain time for early clinical intervention but also reduce medical costs for patients and maximize the benefits of diagnosis and treatment. Therefore, this study developed an effective and reliable HDC nomogram prediction model to understand the basic information and clinical characteristics of hypertension and diabetes among residents of a district in Hongshan, Wuhan.

This study aims to achieve the following objectives:

- (1) To determine the relationship between BMI, WC and WHtR of residents in the jurisdiction and HDC while controlling for as many confounding factors as possible;
- (2) The dose‒response relationship between BMI, WC and WHtR and HDC was described for residents of different sexes and ages.
- (3) Construct the HDC prediction model of district residents based on BMI, WC and WHtR and other factors and obtain the prediction efficiency of BMI, WC and WHtR;
- (4) The direct effect of obesity on the HDC of residents in the jurisdiction was obtained, and the mediating effect size in the relationship between sociodemographic factors, lifestyle and HDC was obtained.

This study is expected to explore the relationship between BMI, WC, WHtR and HDC among residents of the jurisdiction from multiple perspectives. The results of this study can provide theoretical guidance for the prevention of HDC and encourage people to improve their lifestyle, focus on weight and WC control, and reduce the burden of the disease.

## Patients and methods

### Patients

Ethical approval was obtained for this retrospective analysis. Clinical data on 7,200 hypertension patients in his study were collected at a community health service center in Hongshan District, Wuhan City, from January 2022 to December 2022. All patients were randomly divided into a modeling group and a validation group at a 7:3 ratio. At the same time, the model population was divided into two groups based on whether hypertension and diabetes comorbidities occurred, namely, the comorbidity group and the noncomorbidity group.

Baseline clinical data included questionnaires, physical examinations, and clinical tests. The questionnaire includes:①Basic information, including gender, age, education level, marital status, occupation type, family type, etc.② Behavior and lifestyle: including inhaling (active or passive smoking), alcohol consumption, labor intensity, physical exercise, daily static behavior time (including sitting down to work, study, reading, watching TV, using the computer, rest and other static behavior time, but not including sleeping time), salt consumption, oil consumption, etc. ③Knowledge related to chronic diseases: excess salt will affect health, the standard of salt intake per person per day (< 6 g), the standard of edible oil intake per person per day (< 25 g), understanding the characteristics of people at high risk of chronic diseases (people at high risk of chronic diseases are those with one of the following characteristics: blood pressure of 130–139/85–89 mmHg; ​current smoker; ​fasting blood glucose was 6.1 ≤ EBG < 7.0 mmol/L; ​serum total cholesterol was 5.2 ≤ TB < 6.2 mmol/L; ​male WC ≥ 90 cm, female WC ≥ 85 cm); ​chronic illness, family history, etc.

### Survey methods

- 1. Questionnaire survey: All staff received standardized training and conducted face-to-face questionnaire surveys on subjects one by one, including basic sociological information such as gender, age, marital status, education level and household registration.
- 2. Measurements of blood pressure: Three investigators monitored the subjects' blood pressure in a resting state for three consecutive sessions, and the mean blood pressure over the three sessions was taken as the final blood pressure.
- 3. Measurements of glucose and lipids: The subjects were given nonsmoking alcohol and a high-sugar, high-fat diet for 24 h, and after eight hours of fasting, venous blood from the elbow was collected. Blood samples are tested as soon as they are sent to the laboratory on the same day.
- 4. Measurement of WC: According to the method recommended by the World Health Organization (WHO), the measurement is carried out using a standard tape measure that is nonelastic, anti-tensile, and does not deform when subjected to gravity within 100 g. Half of the connecting length between the lower edge of the 12th rib in the middle auxiliary line and the iliac crest was taken as the measuring point, and the measuring edge of the tape measure passed the measuring point on both sides of the body of the study object to measure WC.

### Diagnostic criteria and definitions

#### **Diagnostic criteria for hypertension** [13]

The blood pressure was monitored 3 times on different days without taking antihypertensive drugs, and the diastolic blood pressure was ≥ 90 mmHg (1 mmHg = 0.133 kPa) or systolic blood pressure was ≥ 140 mmHg each time or a previously diagnosed high blood pressure.

The diagnostic criteria for diabetes were as follows [14]: fasting blood glucose ≥ 7.0 mmol/L, 2-h postprandial blood glucose ≥ 11.1 mmol/L, or previously diagnosed diabetes.

Diagnostic criteria for dyslipidemia were as follows [15]: serum total cholesterol (TC) ≥ 5.2 mmol/L, elevated density lipoprotein cholesterol (HDL-C) < 1.0 mmol/L, low-density lipoprotein cholesterol (LDL-C) ≥ 3.4 mmol/L, triglyceride (TG) ≥ 1.7 mmol/L, or previously diagnosed dyslipidemia. Taking lipid-regulating drugs, body mass index (BMI): ≤ 18 is wasting, 18.5 ~ 23.9 is normal, 24 ~ 27.9 is overweight, ≥ 2 is obese; ​according to the criteria for judging metabolic syndrome recommended by the Diabetes Society of the Chinese Medical Association in 2013, WC of men ≧90 cm and WC of women ≧85 cm were judged as abnormal WC (Diabetes Society of the Chinese Medical Association, 2014).

### Data reduction

After completing the questionnaire, Epidata3.1 was used to build the database. The data were input via double-check and processed in SPSS19.0. Based on the values for height and WC, the value of WHtR and its 25th percentile (P25), 50th percentile (P50), and 75th percentile (P75) were calculated.

### Statistical analyses

SPSS 26.0 and R 4.1.3 software were used for statistical analysis. All data are presented as categorical variables, and logistic regression was used for both univariate and multivariate analyses. Subjects included in the study were randomly divided into modeling and validation groups at a 7:3 ratio. ​Variables were initially screened by a single-factor analysis and LASSO regression, and then the predicted variables were finally included in the model by a multifactor regression analysis to construct the columnwise model. The nomogram model was evaluated in terms of differentiation, calibration and clinical net benefit. The differentiation was evaluated by the C-index, receiver operator characteristic (ROC) curve and area under the curve (AUC). The degree of calibration was assessed by the calibration curve. Clinical benefit was assessed by decision curve analysis. *P* < 0.05 was considered statistically significant.

## Results

### Clinical characteristics

In this study, the SPSS statistical software package was used to perform unbiased randomization on 7200 samples. ​The specific operations are as follows: The software package automatically obtains the formula "partition variable" = 2*re.bernoulli(0.6)-1 according to the initializing value of the activity generator, sets it to the probability parameter 0.6 of the Bernoulli variable and assigns the partition value of 1/-1. The partition value of "1" can allocate 70% of the samples (5040 cases); ​the partition value of "-1" allocates 30% of the sample (2160 cases). Bernoulli (0.3) resets the partition variable according to the formula "partition variable" = "partition variable" -re.bernoulli(0.3). Twenty percent (1008 cases) of the partition value "1" is set as the test sample with the value "0". The sample value of 80% (4032 cases) is still "1", which is set as the training sample. A total of 2160 cases of the original partition value "-1" are set as persistent samples, as shown in Table 1.

**Table 1:** Unbiased random distribution of 7200 samples

	**Modeling group**		**Validation group**
	Training sample	Test sample	Adherence sample
**Sample size**	4032	1008	2160
**Percentage**	56%	14%	30%

There were 5040 cases in the modeling group and 2160 cases in the verification group, and there was no statistical significance in clinical data between the two groups (*P* > 0.05, as shown in Table 2).

**Table 2:** Comparison of clinical data between modeling groups and validation groups

Characteristic	Modeling group (*n* = 5040)	Validation group (*n* = 2160)	*t/Z*/χ^2^	*P*
**Gender** [***n*** **(%)]**			1.921^a^	0.166
**Male**	2559(50.77%)	1091 (50.51%)	-	-
**Female**	2481(49.23%)	1069 (49.49%)		
**Age (year)**			1.919	0.055
**35-**	1328(26.35%)	555 (25.69%)	-	-
**≥ 60**	3712(73.65)	1605 (74.31%)		
**Marital status**			1.901	0.073
**Unmarried**	35(0.69%)	12 (0.56%)	-	-
**Married**	4729(93.83%)	2019 (93.47%)		
**Divorced or widowed**	276(5.48%)	129 (5.97%)		
**Educational level**			1.906	0.068
**Primary and below**	2585(51.29%)	1105 (51.16%)	-	-
**Junior high school**	2354(46.71%)	1011 (46.81%)		
**High school**	73(1.45%)	29 (1.34%)		
**Junior college or above**	28(0.55%)	15 (0.69%)		
**Household category**			1.825	0.037
**Nonrural**	530(10.52%)	237 (10.97%)	-	-
**Rural**	4510(89.48%)	1923 (89.03%)		

### Univariate analysis of diabetes comorbidity

**Fig. 1:**
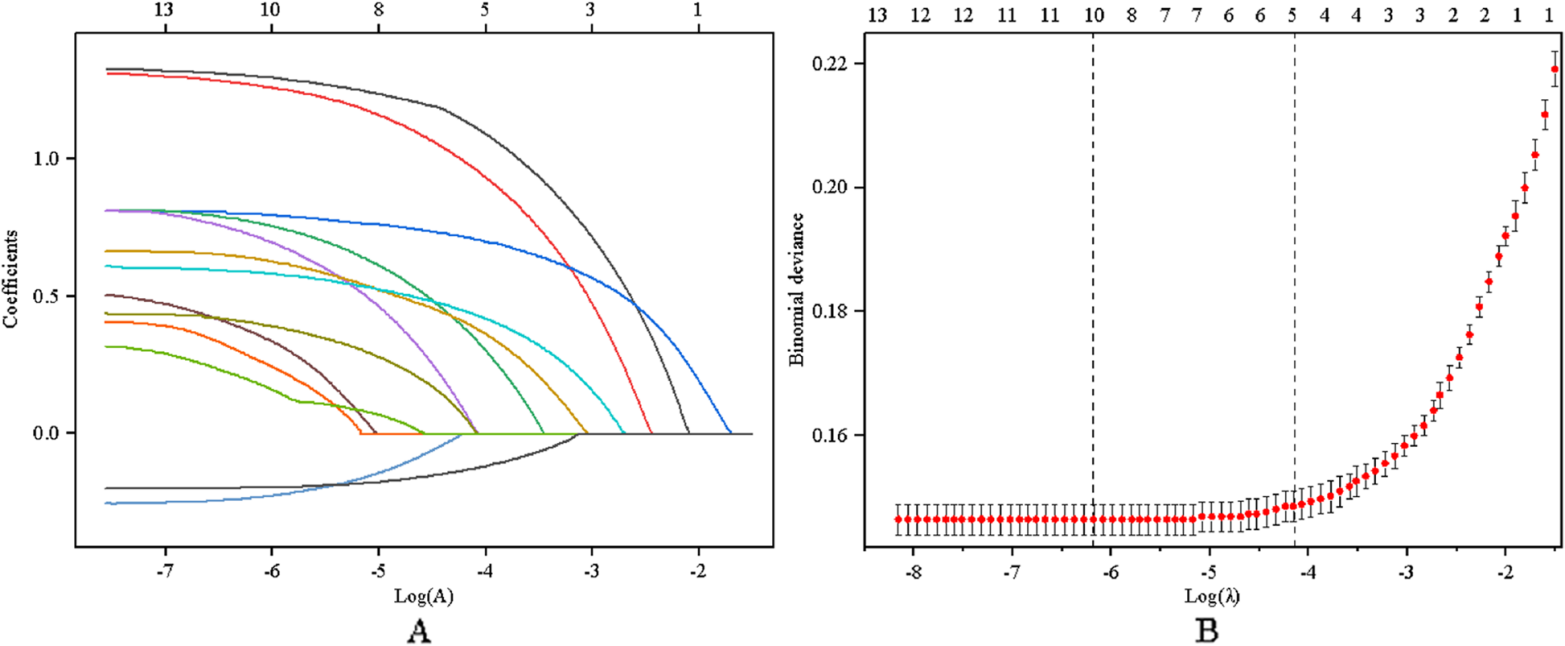
Optimal parameter selection in the LASSO model. **A** LASSO coefficient distribution for all risk factors; **B** Optimal parameter selection in the LASSO model

In the modeling group, the subjects were divided into an HDC group (672 cases) and a non-HDC group (4368 cases) according to whether they had hypertension and diabetes. Through univariate analysis, it was found that sex, age, marriage, education level, family category, BMI, static behavior time, TG, TC, HDL, LDL, WHtR, WC and other differences between the two groups were statistically significant (*P* < 0.05), as shown in Table 3. ​Because of the many risk factors included in this study and the possibility of multicultural relationships among different risk factors, the predictive variables for HDC were selected by LASSO regression analysis. Through LASSO regression model analysis (left of Fig. 1), all variables included in the model are gradually compressed with the change in penalty coefficient λ. To select the least variables for the model and avoid overfitting, 1SE (λ = 0.014), the minimum value in the tenfold cross-validation method, is selected as the optimal value (right of Fig. 1). Five nonzero coefficient predictors were selected from 13 variables, including age, education level, waist-to-hip ratio, family class, and HDL-C.

**Table 3:** Comparison of clinical data between the HDC group and the non-HDC group

Characteristics	HDCgroup (*n* = 672)	Non-HDC group (*n* = 4368)	*χ2*	*P*
**Age(year)**			3.761	< 0.01
**35–60**	85 (12.65%)	2045 (46.82%)		
**≥ 60**	587 (87.35%)	2323 (53.18%)	-	-
**Gender**			54.025	< 0.01
**Female**	305(45.39%)	2585(59.18%)		
**Male**	367(54.61%)	1783(40.82%)		
**Marital status**			191.61	< 0.01
**Unmarried**	72 (10.71%)	340 (7.78%)		
**Married**	457 (68.01%)	3695 (84.59%)		
**Divorced or widowed**	143 (21.28%)	3337.62%)		
**Educational level**			2.958	< 0.01
**Primary and below**	289 (43.01%)	1349 (30.88%)		
**Junior high school**	211 (31.40%)	1190 (27.24%)		
**High school**	118 (17.56%)	952 (21.79%)		
**Junior college or above**	72 (10.71%)	875 (20.03%)		
**Household category**			3.762	< 0.01
**Nonrural**	511 (76.04%)	2540 (58.15%)	-	-
**Rural**	160 (23.81%)	1795 (41.09%)		
**BMI**			127.578	< 0.01
**< 18. 5**	34(5.06%)	473(10.83%)		
**18. 5 ~ 23. 9**	93(13.84%)	317(7.26%)		
**24. 0 ~ 28. 0**	277(41.22%)	2423(55.47%)		
**> 28. 0**	268(39.88%)	1155(26.44%)		
**TG(mmol/L)**			82.878	< 0.01
**< 1.7**	57 (8.48%)	858 (19.64%)	(19.64%)	
**≥ 1.7**	615 (91.52%)	3510 (80.36%)		
**TC(mmol/L)**			46.751	< 0.01
**< 5.2**	74 (11.01%)	916 (20.97%)		
**≥ 5.2**	598 (88.99%)	3452 (79.03%)		
**HDL-C (mmol/L)**			50.251	< 0.01
**≥ 1.0**	75 (11.16%)	930 (21.29%)		
**< 1.0**	597 (88.84%)	3438 (78.71%)		
**LDL-C (mmol/L)**			18.955	< 0.01
**< 3.4**	73 (10.86%)	874 (20.01%)		
**≥ 3.4**	599 (89.14%)	3494 (79.99%)		
**Static action time**			35.723	< 0.01
**< 4**	49 (7.29%)	577 (13.21%)		
**4 ~ < 8**	414 (61.61%)	2329 (53.32%)		
**≥ 8**	209 (31.10%)	1462 (33.47%)		
**WHtR range**			417.81	< 0.01
**< P25**	54 (8.04%)	1127 (25.80%)		
**P25 ~ < P50**	68 (10.12%)	1105 (25.30%)		
**P50 ~ < P75**	190 (28.27%)	1127 (25.80%)		
**P75**	312 (46.43%)	1009 (23.10%)		
**WC**			276.58	< 0.01
**Normal standards**	152 (22.62%)	2367 (60.37%)		
**Beyond the normal standard range**	520 (77.38%)	2001 (45.81%)

### Multivariate analysis of diabetes comorbidity

With the diagnosis of diabetes comorbidity as the dependent variable and the five predictors screened by LASSO regression as the independent variables, the multivariate regression analysis showed that age, WHtR and HDL-C were independent risk factors for hypertension-diabetes comorbidity (*P* < 0.05), as shown in Table 4. ​It is worth noting that we introduce the profile penalized log-likelihood function as a loss function in LASSO regression to remove bias in the odds ratio estimates and improve the accuracy and reliability of the results.

**Table 4:** Results of the multifactor analysis of the comorbidity risk of hypertension and diabetes after modification based on the log likelihood of the profile penalty

Intercept and variable	Prediction model		
	β	Odds ratio (95% CI)	*P*
**Age(year)**	-3.9212	0.0198(0.0085–0.0404)	< 0.001
**WHtR**	1.2848	3.6140(1.5696–9.1761)	< 0.001
**HDL-C**	2.3156	10.1307(4.1368–18.6112)	< 0.001
**Household category**	0.8557	2.3531(0.9656–6.0081)	0.0556
**Educational level**	0.2794	1.3077(0.4194–4.0726)	0.6275

*Β* Regression coefficient, *CI* Confidence interval

Profile penalized log likelihood is a statistical method used to address the inflation of the odds ratio in retrospective studies [16]. This approach adjusts for the bias in effect estimation observed in retrospective studies by modifying the likelihood function. Specifically, the method uses a profile penalized log-likelihood based on a probability distribution. ​By penalizing the likelihood function, this approach can effectively reduce the estimated odds ratio and the width of the confidence interval. This penalty can remove the bias in the odds ratio estimates and improve the accuracy and reliability of the results. In retrospective studies, odds ratio inflation is usually caused by factors such as small sample size, rare events, or special conditions of the data set. The profile penalized log-likelihood method can correct for this inflation and provide a more accurate estimate of the effect.

### ​Establishment and validation of a nomogram model for hypertension risk

**Fig. 2:**
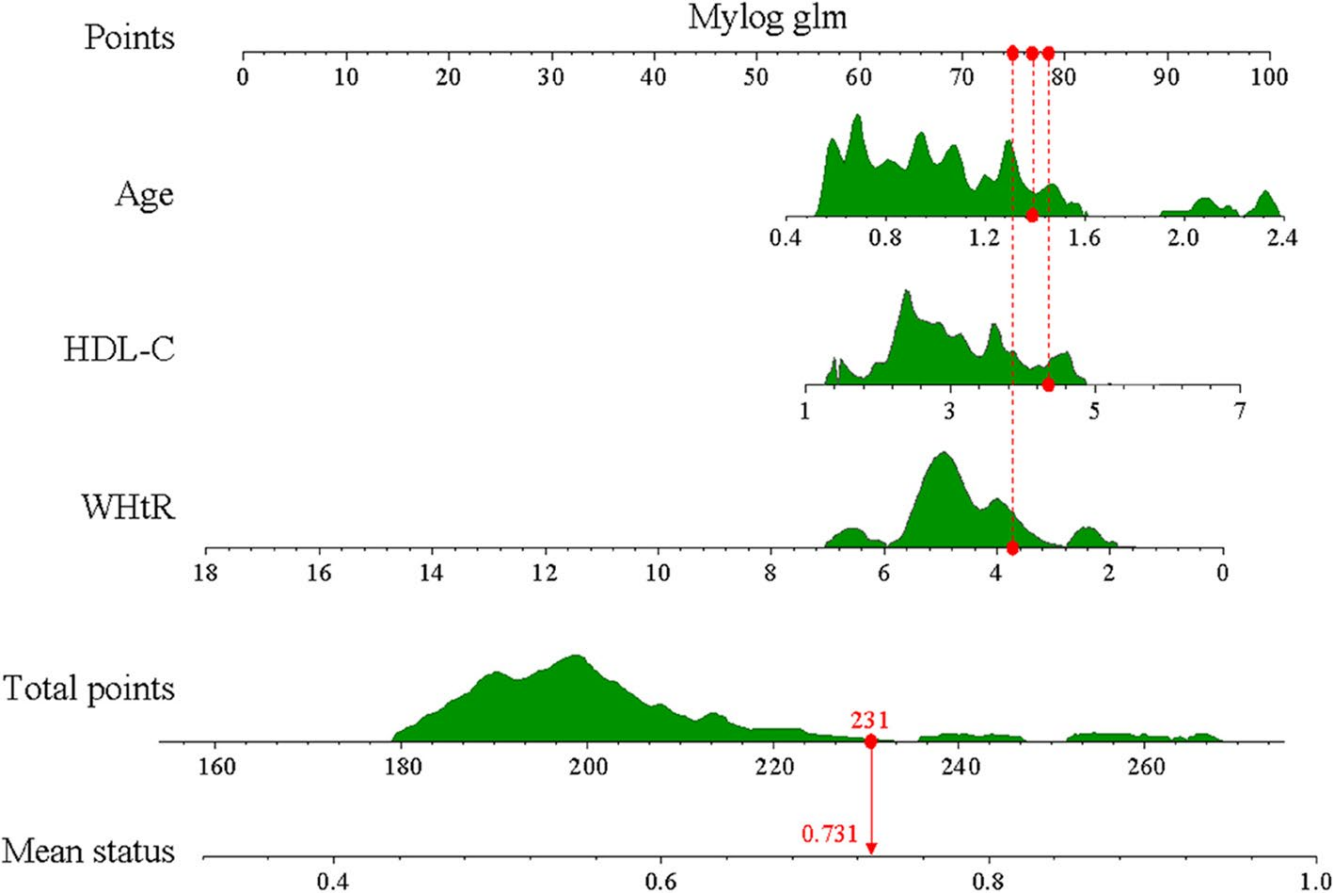
Individualized nomogram prediction models for HDC risk in hypertensive patients and diabetic patients.

The 3 predictors were identified using R software based on multifactor regression analysis, and the hypertension-diabetes comorbidity risk profile was established as shown in Fig. 2.

​Each predictor can be scored according to the top scoring scale via a bar chart. The scores of all predictors can be added together to compute the total score, and a vertical line can be drawn downward according to the total score to obtain the estimated probability of hypertension and diabetes comorbidity. The model differentiation was evaluated by computing the C index and plotting the ROC curve of the nominal model for predicting diabetes comorbidities. The C index of the modeling group and verification group was 0.8585 (0.845 6–0.8714) and 0.8530 (0.8328 ~ 0.8732), respectively.

**Fig. 3:**
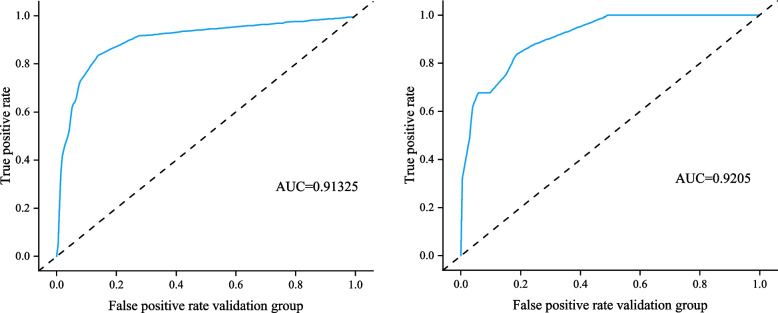
ROC of the predicted model in the modeling and validation populations

The AUC of the modeling group was 0.91325 (95% CI: 0.8572–0.9403) (left of Fig. 3), the AUC of the verification group was 0.9205 (95% CI: 0.8471–0.9527) (right of Fig. 3), and the AUC values were all greater than 0.9, indicating that the model had superior prediction ability.

**Fig. 4:**
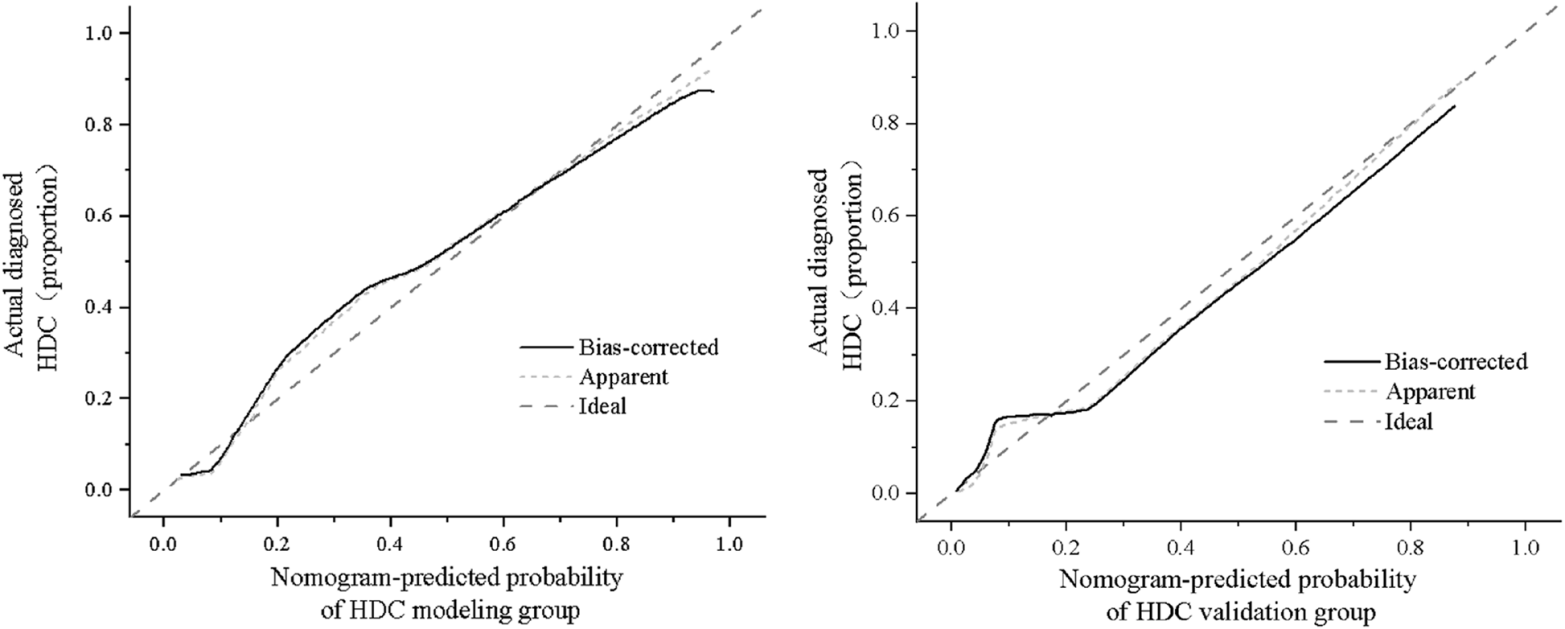
Calibration curves of the prediction model in the modeling and validation populations

The calibration diagram shows that the mean absolute errors (MAEs) of the modeling group (left of Fig. 4) and the verification group (right of Fig. 4) are 0.0215 and 0.0261, respectively, and the mean square errors (MSEs) are 0.0013 and 0.0012, respectively. The calibration curve was close to the standard curve, indicating the acceptable stability of the model in clinical prediction.

**Fig. 5:**
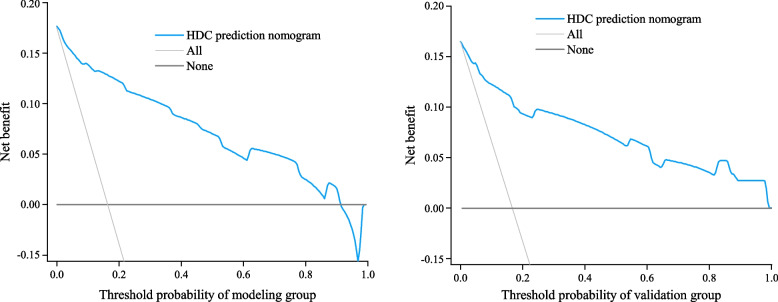
Decision curve of the prediction model in the modeling and validation populations

The clinical benefit of this model was evaluated by DCA. The DCA for the modeling and validation groups are shown on the left and right of Fig. 5 for predicting the risk of hypertension-diabetes comorbidities. The net benefit of using this column to predict the risk of hypertension-diabetes comorbidity is higher when the threshold probabilities in the population decision curves for the modeling and validation groups are 4 − 91% and 2 − 99%, respectively. This indicates that the predictive model has good clinical performance.

## Discussion

​We developed and validated a clinical model for predicting hypertension-diabetes comorbidities for the community management of chronic diseases, which is composed of clinical risk factors readily available in community management. First, 13 clinical risk indicators were identified as independent risk factors through a single-factor analysis of 22 clinical features. ​Due to the possible multicollinear relationship between these risk factors, LASSO regression analysis was used to screen the five predictors with nonzero coefficients from the 13 variables as independent variables for multifactor regression analysis, resulting in three predictors as independent risk factors. The above independent risk factors were then applied to construct a predictive model, which was visualized by constructing a nomogram. The results demonstrate that the established predictive model is well stabilized in both the modeling and validation groups and that the clinical trial nomogram has the potential to be used as a chronic disease management tool for predicting hypertension-diabetes comorbidities in the community.

In terms of clinical features, we found that age and waist-height ratio were independent risk factors for hypertension-diabetes comorbidity, which is also consistent with numerous other findings [17–19]. Among the three potential clinical risk factors, HDL-C level has always been considered a clinical indicator closely related to hypertensive-diabetes comorbidity [17]. This study has shown that low HDL-C levels are an influential predictor of codeveloping diabetes in patients with hypertension, and the reason why HDL-C can reduce the risk of diabetes may be that HDL-C can activate the AMPK (AMP-activated protein kinase) signaling pathway in fat, skeletal muscle and calories, thereby promoting glucose uptake in various peripheral tissues. In vitro experiments have shown that HDL-C can inhibit oxidative stress-induced apoptosis of islet B cells [18]. The functional deficiency of HDL-C can lead to increased sensitivity of islet B cells to oxidative stress and lead to islet inflammation and cholesterol accumulation [19]. At the same time, this study also found that the internal change trend of HDL-C levels over time was slightly different among different individuals, which also revealed that predicting the occurrence of diabetes comorbidity based only on the HDL-C level at baseline or at a certain point may ignore the influence of the continuous change trajectory of HDL-C on diabetes comorbidity. ​The above findings broaden our thinking for additional studies at a later stage. Among the hypertensive population in China, the incidence of dyslipidemia is as high as 42.7%. However, only 20% of patients receive timely clinical treatment for dyslipidemia. The main reason is that health managers do not have an adequate understanding of the correlation between talent and chronic disease progression and do not have an active awareness of monitoring blood lipids in routine chronic disease management. Therefore, we need to not only strengthen normal health education in high-risk groups with dyslipidemia but also monitor the dynamic changes in blood lipids and implement effective lipid management in a timely manner to reduce the risk of cardiovascular disease.​

​In addition, this study attempted to explore additional clinical information on obesity indicators. Studies in the United States and abroad have evaluated the efficacy of WC and WHtR in the diagnosis of abdominal obesity by comparing their association with cardiovascular disease. Hseih et al. found that WHtR was better than WC in evaluating abdominal obesity in the Japanese population [20]. However, there is a large difference in the distribution of WC between men and women [21]. ​Therefore, it is appropriate and reasonable to include BMI, WC and WHtR in the selection of obesity assessment metrics in this study. We utilized the minimal absolute shrinkage and selection operator (LASSO) regression method and found no statistical correlation between elevated WC and HDC. This is at odds with the conclusions of previous studies. Perhaps the most important reason for this discrepancy is that both WHtR and WC are included as independent variables in the analysis. The two independent variables, WC and WHtR, are correlated, and the degree of correlation between them reaches the multicollinearity criterion. Another possible reason is that the nodal splitting of normal and abnormal WC in this study is different from other domestic and international studies. This suggests the importance of obesity control in HDC prevention interventions, which can be based on demographic and sociological characteristics of the population, health knowledge, lifestyle and other targeted measures.

### Study strengths and limitations

This study not only analyzed the basic sociological information of the patients, but also explored the characteristics of the test omics, so as to obtain a simple and easily available predictor. In this study, five nonzero coefficient predictors were selected from 13 selected variables using the minimally absolute shrinkage and selection operator regression method. Moreover, the nonlinear dose–response relationship between WHtR and HDC was found in this study. In the elderly population, the intensity of the association between HDC and WHtR initially increased with the increase of WHtR, but this trend was not unchanged, showing a certain "saturation effect". To facilitate clinical application, this study constructs a clinical test-omics nomogram that combines test-omics features with available clinical features.

The limitations of this study are as follows: (1) The results of this study come from a single institution; therefore, multicenter verification is needed to expand the universality of the experimental results. (2) This study is a retrospective study, and there may be inevitable selection bias. In summary, this study establishes a clinical-lab nomogram that can be used as a clinical tool for individualized hypertension-diabetes comorbidities to assist clinical treatment decision-making and enable precision medicine.

## Conclusions

Hypertension and diabetes are mutually progressive, and both are closely linked to insulin resistance. To facilitate the management of chronic diseases in the community, a simple and accessible clinical-laboratory nomogram was constructed. With this scoring system, the risk of comorbidity in hypertensive patients is generated, allowing grassroots clinicians to individually predict the risk of comorbidity in hypertension and diabetes, in line with the current trend of individualized precision medicine.

​The high accuracy of the nomogram prediction model for hypertension-diabetes comorbidities developed in this study not only enables early identification and prediction of HDC risk in hypertensive patients but also provides more favorable clinical guidance for grassroots staff and patients in medical interventions and lifestyle monitoring and is worth promoting.​

## Data Availability

​Data for the current study are available from the corresponding author upon reasonable request.
